# Lack of utility of phosphate serum monitoring in HIV-infected patients on a tenofovir-based antiretroviral regimen

**DOI:** 10.1186/1758-2652-13-S4-P150

**Published:** 2010-11-08

**Authors:** P Wikman, P Safont, MJ Perez-Elías, A Moreno, F Dronda, S Moreno, JL Casado

**Affiliations:** 1 Hospital Ramón y Cajal, Ctra. Colmenar Viejo Km. 9100, Madrid, Spain

## Purpose of the study

Hypophosphatemia (HP) and renal dysfunction have been associated with antiretroviral therapy, especially with the use of tenofovir disopropil fumarate (TDF). Thus, recent guidelines recommend routine phosphate measurements in HIV-infected patients. We aimed to assess the utility of this monitoring.

## Methods

Retrospective cohort study of 680 patients with renal monitoring on antiretroviral therapy from 2008 to 2010. The frequency of HP was compared in TDF recipients with that in non-TDF recipients, as well as assessed the reproducibility of HP, and identified the incidence of renal dysfunction in hypophosphatemic patients

## Results

Phosphate measurements were obtained in 265 patients during follow-up. Mean age was 40.66 (±7.68) years, 76.2% were men, 47.9% were IDU, and 84.9 % received an antiretroviral regimen based on TDF. At baseline, before antiretroviral therapy, hypophosphatemia was observed in 4 of 67 patients (6%). Overall, during follow up, HP was observed in 56 of 265 (21.1%), but was confirmed only in 33 (12.5%). The median time to HP was 798(±13.95) days, usually with phosphate levels above 2 mg/dl (mild HP). A higher percent of patients prescribed TDF showed a phosphate measurement below normal limits (13.8%) in comparison with those patients receiving non-TDF based therapy (5%), although this comparison was not significant (p=0.103). There was no difference with regard to time to HP in patients receiving TDF or not (median time 798 vs 834 days, p=0.106 log-rank test), neither in the values of serum creatinine or MDRD in patients with or without hypophosphatemia.

## Conclusions

HP is relatively frequent in HIV infected patients on antiretroviral therapy, although we did not find any clear association with any specific therapy or renal dysfunction as measured by serum creatinine or glomerular filtration. This fact questions the utility of routine phosphate testing, in isolation, in TDF recipients.

